# Effect of Natalizumab on Circulating CD4^+^ T-Cells in Multiple Sclerosis

**DOI:** 10.1371/journal.pone.0047578

**Published:** 2012-11-30

**Authors:** Lars Börnsen, Jeppe Romme Christensen, Rikke Ratzer, Annette Bang Oturai, Per Soelberg Sørensen, Helle Bach Søndergaard, Finn Sellebjerg

**Affiliations:** Danish Multiple Sclerosis Center, Department of Neurology, Copenhagen University Hospital Rigshospitalet, Copenhagen, Denmark; University Hospital La Paz, Spain

## Abstract

In multiple sclerosis (MS), treatment with the monoclonal antibody natalizumab effectively reduces the formation of acute lesions in the central nervous system (CNS). Natalizumab binds the integrin very late antigen (VLA)-4, expressed on the surface of immune cells, and inhibits VLA-4 dependent transmigration of circulating immune-cells across the vascular endothelium into the CNS. Recent studies suggested that natalizumab treated MS patients have an increased T-cell pool in the blood compartment which may be selectively enriched in activated T-cells. Proposed causes are sequestration of activated T-cells due to reduced extravasation of activated and pro-inflammatory T-cells or due to induction of VLA-4 mediated co-stimulatory signals by natalizumab. In this study we examined how natalizumab treatment altered the distribution of effector and memory T-cell subsets in the blood compartment and if T-cells in general or myelin-reactive T-cells in particular showed signs of increased immune activation. Furthermore we examined the effects of natalizumab on CD4^+^ T-cell responses to myelin in vitro. Natalizumab-treated MS patients had significantly increased numbers of effector-memory T-cells in the blood. In T-cells from natalizumab-treated MS patients, the expression of TNF-α mRNA was increased whereas the expression of fourteen other effector cytokines or transcription factors was unchanged. Natalizumab-treated MS patients had significantly decreased expression of the co-stimulatory molecule CD134 on CD4^+^CD26^HIGH^ T-cells, in blood, and natalizumab decreased the expression of CD134 on MBP-reactive CD26^HIGH^CD4^+^ T-cells *in vitro*. Otherwise CD4^+^ T-cells from natalizumab-treated and untreated MS patients showed similar responses to MBP. In conclusion natalizumab treatment selectively increased the effector memory T-cell pool but not the activation state of T-cells in the blood compartment. Myelin-reactive T-cells were not selectively increased in natalizumab treated MS.

## Introduction

Relapses in multiple sclerosis (MS) are caused by focal inflammatory lesions, consisting of activated macrophages and CD4^+^ and CD8^+^ T-cells, in the central nervous system (CNS). Binding of the integrin very late antigen (VLA)-4, expressed on the surface of most immune cells, to the vascular cells adhesion molecule (VCAM)-1, expressed on endothelial cells, is important for the adhesion and transmigration of systemically activated immune cells from the blood into the CNS. VLA-4 consists of an α4 (CD49d) and β1 chain (CD29). Besides being an adhesion molecule, VLA-4 has co-stimulatory effects in T-cell activation [1]–[3].

The humanized monoclonal IgG4 antibody natalizumab binds VLA-4, and the use of natalizumab as MS treatment is well established and effective, reducing clinical and MRI measures of disease activity [4]. It is assumed that the primary therapeutic effect of natalizumab relies on an inhibition of VLA-4-mediated extravasation of activated immune cells into the CNS [3]. Still, it is not fully understood if natalizumab inhibits transendothelial migration simply by blocking ligation of VCAM-1 and VLA-4, as decreased levels of VLA-4 have been detected on the T-cell surface in natalizumab-treated MS-patients [1], [5], [6]. This observation could be an artifact due to competitive binding of natalizumab and the antibody used to visualize the VLA-4 molecule for flow cytometry, due to natalizumab induced internalization of VLA-4 or reduction of α4 or β1 integrin chain synthesis.

Previous studies have shown that natalizumab-treated MS patients have an increased pool of T-cells and other lymphocytes in the blood compartment [7]–[9] which may be enriched in T-cells with an increased activation state or an increased potential to produce pro-inflammatory cytokines [10]–[12]. A possible explanation is that pro-inflammatory T-cells may be selectively retained in blood due to VLA-4 blockade [12]. Alternatively natalizumab may interfere with immune and T-cell activation *in vivo* [13], as different studies showed that VLA-4-antibodies may enhance or block co-stimulatory signals provided by VLA-4 in anti-CD3 induced T-cell proliferation and cytokine production [3]. Yet, studies examining whether natalizumab interferes with the antigen-specific activation of CD4^+^ T-cells are rare.

To what extent minor T-cell subsets may be selectively increased in an enriched T-cell pool has so far not been studied in depth [1], [6]. Memory T-cells (CD45RA^−^) can be divided into central memory (CCR7^+^) and effector memory (CCR7^−^) T-cells [14], [15] and may have different roles in the immunopathogenesis of MS [16]. Assessing the CD45RA and CCR7 (CD197) expression by flow-cytometry, T-cells can be divided into: antigen-inexperienced naïve (CCR7^+^CD45RA^+^) T-cells and antigen-experienced memory (CD45RA^−^) T-cells. Memory T-cells can further be characterized by expression of CCR7 (CD197). CCR7 is a homing receptor that enables the central memory (CCR7^+^CD45RA^−^) T cell to enter secondary lymphatic tissue. Upon antigen-specific reactivation, central memory T-cells have a high capacity to proliferate but low capacity to produce effector cytokines. In contrast effector memory T-cells (CCR7^−^CD45RA^−^) are able to produce high levels of effector cytokines but do only show little proliferation upon activation by local APCs in the peripheral tissues. Depending on the expression of CD27 these cells can be further divided into subsets displaying different stages of differentiation [14], [15].

CD26 and CD134 are co-stimulatory molecules which are expressed on T-cells. Increased CD26 expression on T-cells has been associated with disease activity in MS and it has been suggested that CD134 has a pathogenic role in different auto-immune disease [17]–[19]. All major IL-17 producing T-cell subsets express CD161 [20] and a single nucleotide polymorphism of the CD161 gene (*KLRB1*) has been associated with susceptibility to develop MS [21], [22]. The effect of natalizumab on CD26, CD134, CD161 and central-memory and effector-memory T-cells has not been studied yet.

We hypothesized that treatment with natalizumab would result in the retention of effector T cells, myelin reactive T-cells producing pro-inflammatory cytokines and other circulating T-cell subsets that have been associated with MS. These hypotheses were addressed by studying the phenotype of circulating T cell subsets by flow cytometry and analysis of the gene expression, and by assessing CD4^+^ T cell reactivity to myelin basic protein (MBP) in untreated and natalizumab-treated MS patients.

## Methods

### Ethics Statement

The study was approved by the regional scientific Ethics Committee of Copenhagen County (protocol KF 01-0141/95 and KF 01-314009) and all persons participating in this study provided written informed consent.

### Patients

For this study we obtained blood samples from 21 untreated and 25 natalizumab-treated MS patients with a relapsing-remitting disease course. The patients included in this study did not receive treatment with glucocorticoids or other immunomodulatory drugs and did not show signs of a relapse within a period of two months prior to sampling. Among the untreated MS patients, 4 patients had previously received immunomodulatory treatment but stopped for different reasons while the remaining 17 patients were treatment naïve. 14 of the treatment naïve patients were newly diagnosed with MS (within the preceding 12 months) scheduled to start immunomodulatory treatment. The median age was 35 years (IQR = 27–42) in the untreated group and 36 years (IQR = 32–44) in the natalizumab-treated group; the degree of disability was significantly lower among untreated MS patients (median EDSS 1.5; IQR = 1–3) than among natalizumab-treated MS patients (median EDSS 2.5; IQR = 2–4; p = 0.016); the median disease duration was significantly shorter among untreated MS patients (median 2 years; IQR = 1–8 years) than among natalizumab-treated MS-patients (median 9 years; IQR = 6–12 years; p = 0.001). The effect of natalizumab treatment on the mRNA expression of genes encoding the VLA-4 subunits α4 (*ITGA4*) and β1 (*ITGB1*), was measured in whole blood samples from 25 untreated RRMS patients and from 50 RRMS patients 3, 6 and 12 months after initiation of natalizumab treatment.

For PCR analysis of the gene expression in the blood, whole blood was collected in Tempus tubes (Applied Biosystems, USA).For all other studies blood was drawn in BD Vacutainer® EDTA tubes (BD Biosciences, Denmark), and PBMCs were promptly separated by density gradient centrifugation on Lymphoprep (Axis-Shield, Norway).

### Separation of blood cell subsets

CD4^+^ and CD8^+^ T-cells were separated from freshly isolated PBMCs using CD4^+^ T Cells Isolation Kit II, CD8^+^ T Cells Isolation Kit II and an autoMACS™ cell-separator (all from Miltenyi Biotec, Germany) according to the manufacturer's instructions.

### Gene expression analysis

For quantitative real-time PCR (qPCR) analysis in whole blood cells mRNA was extracted with the Tempus 12-port RNA isolation kit (Invitrogen, Denmark) and cDNA was synthesized with the High Capacity cDNA RT kit (Applied Biosystems). For gene expression analysis of CD4^+^ and CD8^+^ T-cells, mRNA was isolated using Pico Pure RNA Isolation Kits (Arcturus, USA) and cDNA was synthesized with qScript cDNA SuperMix (Quanta BioSciences, USA). qPCR analyses were run on an ABI 7500 Real Time PCR System (Applied Biosystems) using predesigned and validated primers (Applied Biosystems; Table S1). Gene expression in each sample of the target mRNA, relative to an endogenous reference gene (ΔC_t-sample_), was compared to a calibrator consisting of pooled cDNA made from PBMCs from healthy volunteers (ΔC_t-pool_). We used *CASC3* and *UBE2D2* as reference genes. Gene-expression levels are given as normalization ratio (NR) calculated as: NR = 2^−ΔCt(sample)−ΔCt(pool)^ [23].

### Cell culture

52.5×10^6^ freshly isolated PBMCs were stained in 1.5 ml PBS containing 1 µM carboxyfluorescein diacetate succinimidyl ester (CFSE; Molecular Probes, Denmark) for 2.5 minutes at room temperature. After washing, 1.7×10^6^ PBMCs in 714 µl culture medium (CM) were transferred to flat bottom 48-well culture plates (Cellstar®; Greiner bio-one, Germany). As antigens we used tetanus toxoid (TT; 10 µg/ml; (Statens Serum Institut, Copenhagen, Denmark) or myelin basic protein (MBP; 30 µg/ml; HyTest, Finland). For some studies we added natalizumab (25 µg/ml; Biogen Idec, Denmark) or a nonspecific IgG4 control antibody (25 µg/ml; Sigma, Denmark). Cells were incubated for 7 days at 37°C in a humidified 5% CO_2_ atmosphere. To stain intracellular cytokines on day 7, 100 µl of the supernatant was replaced with fresh CM containing 10 ng/ml of phorbol 12-myristate 13-acetate(PMA) and ionomycin 1 mM (both Sigma). After 1 hour, brefeldin A (5 µg/ml; Sigma) was added and the cells were further incubated for 4 hours.

### Flow cytometry analysis of CD4^+^ T-cell reactivity to MBP and TT

For flow cytometry we used a BD FACSCanto II™ and the BD FACSDiva™ Software 6.1.2 (both from BD Biosciences, Denmark). Cells were harvested, washed in PBS at 4°C, and stained with anti-CD3 PacificBlue (PB), anti-CD4-PerCP-Cy5.5, anti-CD8-PeCy7, anti-CD19-APC-Cy7 and dead/live staining dye (Table S2) in a 50 µl reaction for 30 minutes in the dark at 4°C. Then the cells were washed in FACS-PBS (PBS/1% (w/v) HSA/2 nM EDTA (FACS-PBS)) and re-suspended in 100 µl FACS-PBS for flow cytometry. The proliferation of CD4^+^ T-cells was assessed as the percentage of CFSE-diluted cells within the CD3^+^CD4^+^ population.

To measure the intracellular cytokine production in proliferating CD4^+^ T-cells, the cells were stained as described above using anti-CD3-PB, anti-CD8-PeCy7, anti-CD19-APC-Cy7 and live/dead staining dye (Invitrogen, Denmark). Cells were not stained for CD4 as PMA induced a significant down-regulation of CD4 (data not shown). Cells were fixed and permeabilized with the FOXP3 permeabilization kit (BioLegend,USA) and then stained for 30 minutes at room temperature with combinations of: anti-IL-17A-PE and anti-IFN-γ-APC; anti-IL-4-PE and anti-TNF-α-APC; and anti-IL-10-PE and anti-IL13-APC (Table S2). The cytokine expression was measured in proliferating and non-proliferating CD8^−^ and CD8^+^ T-cells using flow cytometry. As control for non-specific background fluorescence and non-specific antibody binding, TT-stimulated cells were stained with isotype controls (Table S2).

### Flow-cytometry of CD4^+^ and CD8^+^ T-cells

Freshly isolated PBMCs were re-suspended in staining buffer (eBiosciences, USA). In a 65 µl reaction 5×10^5^ PBMCs were stained with fluorochrome-conjugated antibodies for the surface markers CD3, CD4, CD8 and CD49d together with combinations of: CD26, CD134 and CD154; CD161, IL23R and CD212; CD11a and CD18; or CCR7, CD45RA and CD27. As control for non-specific antibody binding, non-specific fluorescence and spectral overlap we used the fluorescence minus one method [24] combined with Isotype-matched control antibodies (Table S2). The expression of the stained surface molecules was measured on CD3^+^CD4^+^CD8^−^ and CD3^+^CD4^−^CD8^+^ T-cell subsets by flow cytometry. Depending on the expression pattern of the target molecule, expression levels were assessed as median fluorescence intensity (MFI) or percentage of positively stained cells within a defined subset. To assess absolute numbers of T-cell subsets, 50 µl blood was stained with 20 µl BD Multitest™ antibody cocktail (BD Biosciences), containing antibodies against CD3, CD4, CD8 and CD45 in Trucount™ tubes for 15 min at room temperature followed by red blood cell lysis by adding 450 µl BD FACS lysing solution (BD Biosciences) to the cells for 20 minutes at room temperature. Finally, cells were measured by flow cytometry and counts (cells/µl) of CD3^+^CD4^+^CD8^−^ and CD3^+^CD4^−^CD8^+^ cells were calculated according to the instructions of the Trucount™ manufacturer (BD Biosciences).

### Data and statistics

Data in figures are shown as boxplots; the box represents the interquartile range (IQR); the median value is indicated by a line; the whiskers represent range. Data in tables are given as median with IQR. If not otherwise stated, differences were tested for significance by non-parametric tests: Mann-Whitney U test for unpaired samples; Wilcoxon Signed Ranks test for paired samples. P-values below 0.01 were considered statistically significant. For data processing we used Microsoft Excel and SPSS v.18.

## Results

### Circulating T cells and VLA-4 expression in natalizumab-treated MS

**Figure 1:**
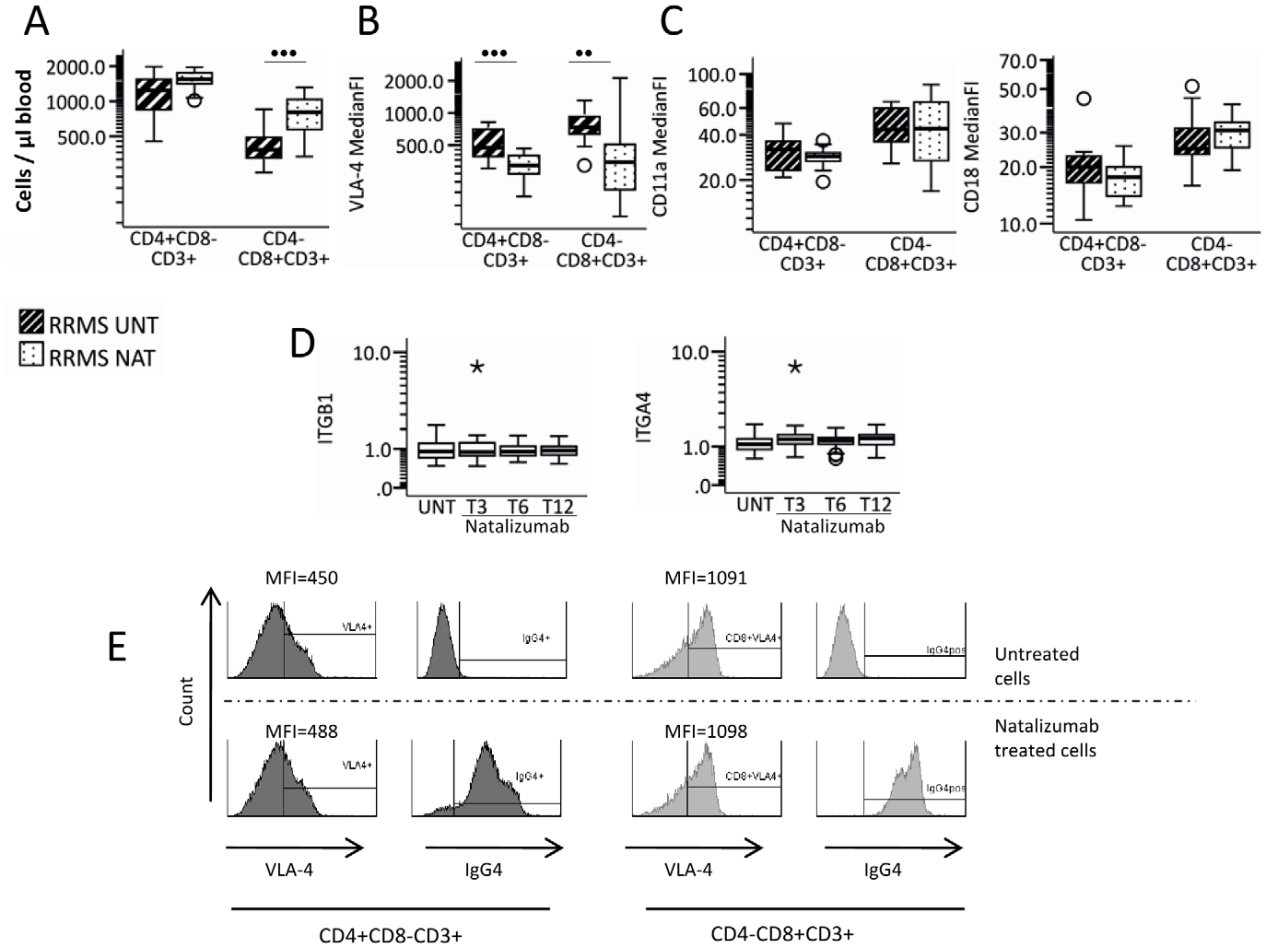
T-cell counts and integrin expression. (A) Number of circulating CD4^+^CD8^−^ and CD4^−^CD8^+^ T-cells and surface expression of (B) VLA-4 and (C) CD11a and CD18 on CD4^+^ and CD8^+^ T-cells (median fluorescence intensity, MFI) from 13 untreated and 17 natalizumab treated RRMS patients. (D) mRNA expression of *ITGB1* and *ITGA4* measured by quantitative real-time PCR in untreated RRMS (UNT; n = 25) and 50 natalizuamb treated RRMS patients 3 months (T3), 6 months (T6) and 12 months (T12) after initiation of treatment. (E) Histograms show the expression of VLA-4 and the surface-binding of IgG4 on CD4^+^CD8^−^ and CD4^−^CD8^+^ T-cells after incubation of PBMCs from a healthy volunteer with and without natalizumab 25 µg/ml in PBS for 1 hour at 4°C. Boxes represent interquartile range, median value indicated as a line, whiskers represent range, ^o^ = outliers, * = extremes.

Numbers of circulating CD8^+^ T-cells were significantly higher in natalizumab-treated than in untreated MS patients; numbers of CD4^+^ T-cells were also higher, although not significantly different (Figure 1A). Natalizumab-treated patients had significantly lower cell surface expression of VLA-4 on CD4^+^ T-cell and CD8^+^ T-cells (Figure 1B). This did not reflect a general decrease in integrin expression since the surface expression of the integrin LFA-1's α-chain (CD11a) and β-chain (CD18) on CD4^+^ T-cells and CD8^+^ T-cells did not differ in untreated and natalizumab-treated MS-patients (Figure 1C). Neither did it appear to be due to repression of the genes encoding the VLA-4 subunits α4 (*ITGA4*) and β1 (*ITGB1*) since the expression of these genes was comparable in whole blood samples from untreated and natalizumab-treated MS patients (Figure 1D). Finally *ex vivo* studies showed that natalizumab did not interfere with the binding of the anti-VLA-4 antibody used in the flow cytometry studies, suggesting that these antibodies bind to different epitopes, thereby validating the use of this antibody for flow cytometry analysis of VLA-4 expression in natalizumab-treated MS patients (Figure 1E). Thus lower VLA-4 expression on CD4^+^ and CD8^+^ T-cells in natalizumab-treated MS patients appears to be due to internalization or shedding of VLA-4 and not decreased synthesis.

### Naïve, central memory and effector memory T-cells

**Figure 2:**
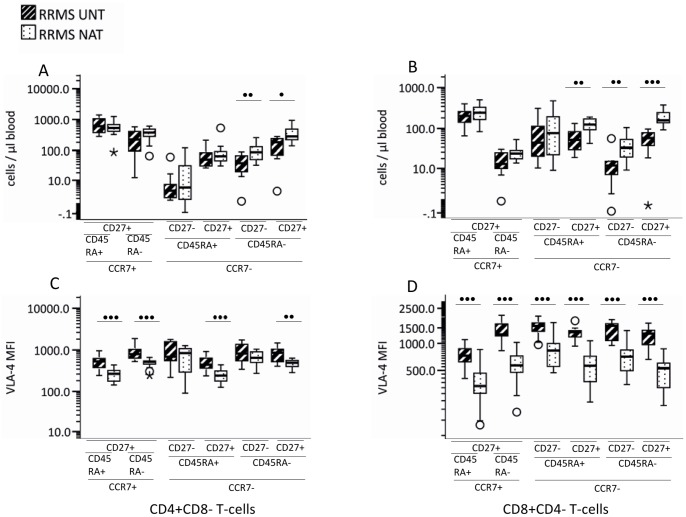
Naïve, memory and effector T-cell subsets in the blood compartment of 13 untreated and 17 natalizumab treated MS patients. Numbers of circulating naïve (CCR7^+^CD45RA^+^CD27^+^), central memory (CCR7^+^CD45RA^−^CD27^+^), effector (CCR7^−^CD45RA^+^CD27^+/−^) and effector memory (CCR7^−^CD45RA^−^CD27^+/−^) (A) CD4^+^ T-cell and (B) CD8^+^ T-cell subsets were assessed by flow cytometry. VLA-4 surface expression on circulating naïve (CCR7^+^CD45RA^+^CD27^+^), central memory (CCR7^+^CD45RA^−^CD27^+^), effector (CCR7^−^CD45RA^+^CD27^+/−^) and effector memory (CCR7^−^CD45RA^−^CD27^+/−^) and (C) CD4^+^ T-cell and (D) CD8^+^ T-cell subsets is given as median fluorescence intensity (MFI). Boxes represent interquartile range, median value indicated as a line, whiskers represent range, ^o^ = outliers, * = extremes. Statistics was by Mann-Whitney U test. • = p<0.01; •• = p<0.005; ••• = p = 0.001. UNT = untreated; NAT = natalizumab treated; NEG = negative; VLA = very late antigen.

Natalizumab-treated patients had significantly increased numbers of circulating effector-memory (CD197^−^CDRA45^−^CD27^+/−^) CD4^+^ and CD8^+^ T-cells (Figure 2A and Figure 2B). The number of CD197^−^CD45RA^+^CD27^+^ CD8^+^ T-cells, a subtype of partially differentiated effector CD8^+^ T-cells was also significantly higher in natalizumab-treated MS-patients (Figure 2B). In natalizumab-treated MS patients, VLA-4 expression was significantly decreased on CD197^+^CD4^+^ T-cells and CD27^+^CD197^−^ CD4^+^ T-cells but not on CD27^−^ CCR7^−^ CD4^+^ T-cells (Figure 2C). VLA-4 expression was significantly decreased on all CD27, CD45RA and CD197-defined CD8^+^ T-cell subsets from natalizumab treated patients.

The natalizumab-treated MS patients had a significantly higher EDSS and significantly longer disease duration than the untreated MS patients; but calculating the Spearman rank correlation coefficient (SRCC) we did not find any association of EDSS or disease duration with the count of circulating naïve, central-memory, effector-memory or effector T-cells (data not shown).

### CD161, IL23R and CD212 expression

**Figure 3:**
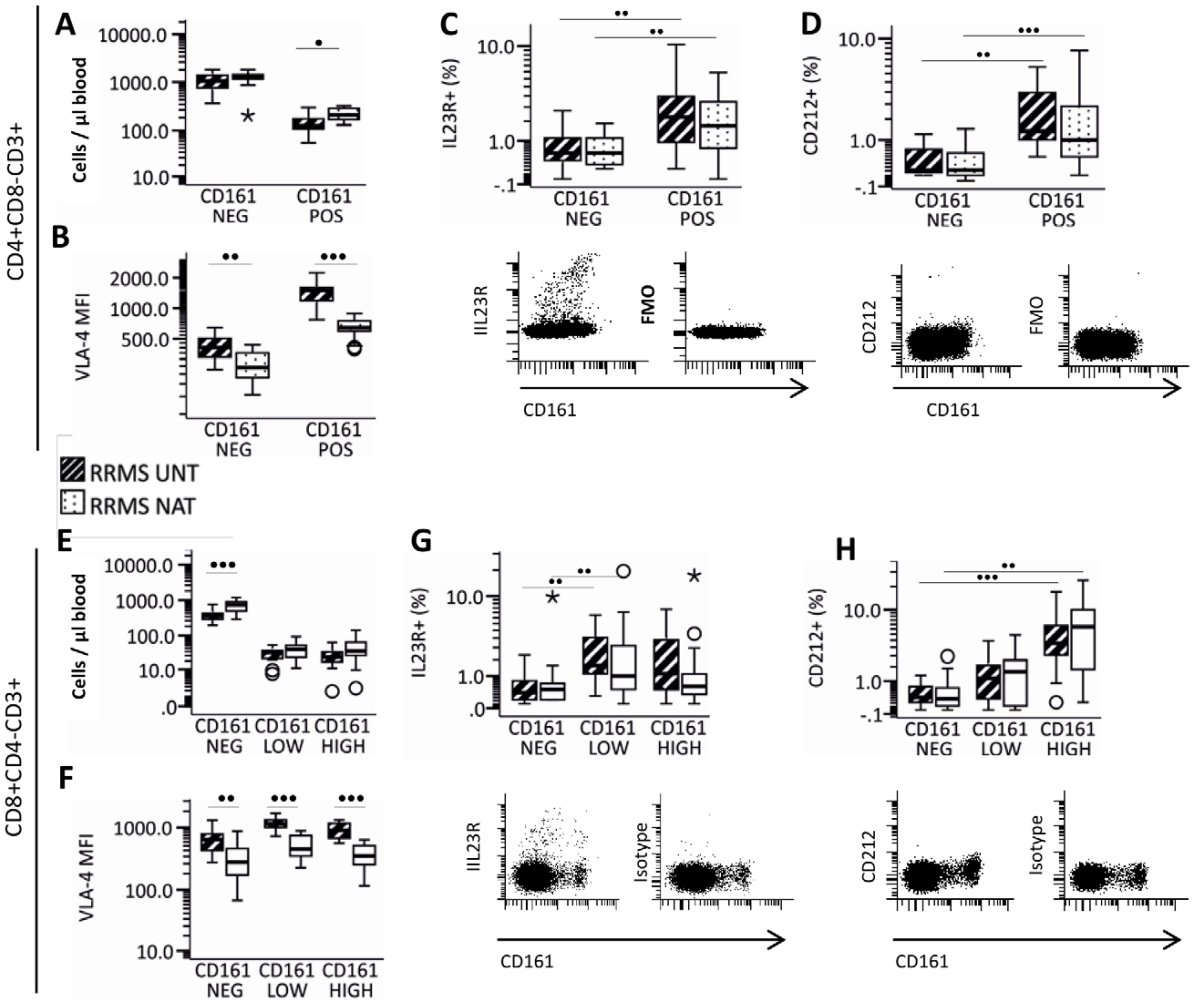
Interleukin (IL)-23 receptor (R), IL12 receptor (CD212), and VLA-4 on CD161 defined subsets of CD4^+^ or CD8^+^ T-cells from 13 untreated and 17 natalizumab treated MS patients. Concentration of (A) CD161^NEG^ and CD161^POS^ CD4^+^ T-cells and (E) CD161^NEG^, CD161^LOW^ and CD161^HIGH^ CD8^+^ T-cells in the blood. VLA-4 expression on CD161 defined (B) CD4^+^ or (F) CD8^+^ T-cell subsets is given as median fluorescence intensity (MFI). IL23R and CD212 expression was assessed as percentage of positive cells within CD161-defined (C and D) CD4^+^ or (G and H) CD8^+^ T-cell subsets, afterwards the unspecific antibody staining, assessed in the corresponding combined isotype and fluorescence-minus one control , was subtracted. Boxes represent interquartile range, median value indicated as a line, whiskers represent range, ^o^ = outliers, * = extremes. Statistics was by Mann-Whitney U test. • = p<0.01; •• = p<0.005; ••• = p = 0.001.

By flow-cytometry we were able to distinguish two CD161-defined subpopulations of CD4^+^T-cells (CD161^−^ and CD161^+^) and three CD161-defined subpopulations of CD8^+^ T cells (CD161^NEG^, CD161^LOW^ and CD161^HIGH^) (Figure 3). There were significantly higher numbers of circulating CD161^+^ CD4^+^ T-cells and CD161^NEG^CD8^+^ T-cells in natalizumab-treated than in untreated MS patients (Figure 3A and Figure 3E). In natalizumab-treated MS patients, VLA-4 expression was significantly decreased on all CD161-defined CD4^+^ and CD8^+^ T-cell subsets (Figure 3B and Figure 3F). In untreated and natalizumab-treated MS patients, significantly more CD161^+^CD4^+^ T-cells and CD161^INT^CD8+ T-cells expressed IL23R as compared to CD161^−^ CD4^+^ and CD8^+^ T-cells (Figure 3C and Figure 3G); and significantly more CD161^+^CD4^+^ T-cells and CD161^HIGH^CD8+ T-cells expressed CD212 as compared to CD161^−^ CD4^+^ and CD8^+^ T-cells (Figure 3D and Figure 3H). The expression of IL-23R, CD212 and CD161 on CD4^+^ and CD8^+^ T-cells was, however, not different in untreated and natalizumab-treated MS patients.

### CD26, CD134 and CD154 expression

**Figure 4:**
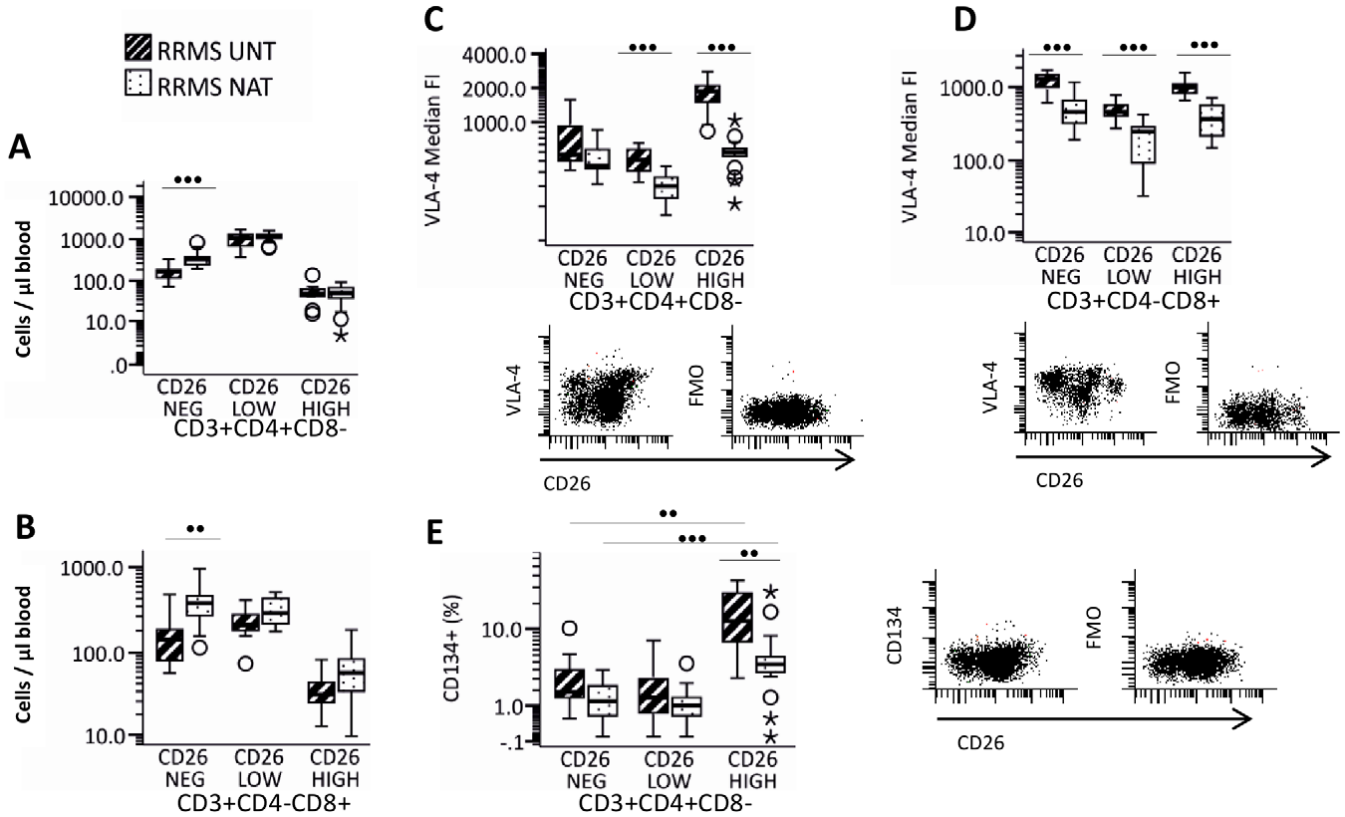
CD134 and VLA-4 on CD26-defined subsets of CD4^+^ or CD8^+^ T-cells from 13 untreated and 17 natalizumab treated MS patients. Concentration of CD26 defined CD4^+^ T-cell and CD8^+^ T-cell subsets in the blood. VLA-4 expression on CD26-defined (C) CD4^+^ or (D) CD8^+^ T-cell subsets is given as median fluorescence intensity (MFI) and representative dot-plots used for analysis are shown. (E) CD134 expression was assessed as percentage of positive cells within CD26 defined CD4^+^ T-cells subsets and afterwards the background-staining, assessed in the corresponding combined isotype and fluorescence-minus one control, was subtracted. CD8^+^ T-cell subsets did not stain positive for CD134 and are not shown here. Boxes represent interquartile range, median value indicated as a line, whiskers represent range, ^o^ = outliers, * = extremes. Statistics was by Mann-Whitney U test, for comparison between the cohorts, and by Wilcoxon's test for comparrison of paired parameters. •• = p<0.005; ••• = p = 0.001. NEG = negative; VLA = very late antigen.

Natalizumab-treated patients had significantly higher numbers of circulating CD26^NEG^CD4^+^ T-cells and CD26^NEG^CD8^+^ T-cells as compared to untreated MS-patients (Figure 4A and Figure 4B). Except for CD26^NEG^CD4^+^ T-cells, VLA-4 expression was significantly decreased on all CD26-defined CD4^+^ and CD8^+^ T-cells subsets in natalizumab-treated patients as compared to untreated RRMS (Figure 4C and Figure 4D) patients. Globally, CD134 expression on CD4^+^ T-cells was not significantly different in untreated and natalizumab-treated MS-patients (data not shown), but the percentage of CD26^HIGH^CD4^+^ T-cells which expressed CD134 was significantly lower in natalizumab-treated than in untreated MS patients (Figure 4E). We did not find any association of disease duration or EDSS with the percentage of CD26^HIGH^CD4^+^ T-cells which expressed CD134 (correlation analyzed by the SRCC; data not shown). CD8^+^T-cells did not stain positive for CD134 and CD4^+^T-cells and CD8^+^T-cells did not stain positive for CD154 (data not shown).

### Gene expression in CD4^+^ and CD8^+^ T-cells in natalizumab treated MS

Natalizumab-treated MS patients had significantly increased expression of *TNF* and significantly decreased expression of *IL10* in circulating CD4^+^ and CD8^+^ T-cells (Table 1). We did not find any correlation between EDSS or disease duration with the mRNA expression of *TNFA* or *IL10* in CD4^+^ and CD8^+^ T-cells (correlation analyzed by the SRCC; data not shown). The expression of *TBX21*, *HLX1*, *IFNG*, *RORC*, *IL17A*, *IL17F*, *IL22*, *GATA3*, *IL4*, *IL5*, *FOXP3*, *TGFB1*, *PRF1* in circulating CD4^+^ and CD8^+^ T-cells was not different, comparing untreated and natalizumab-treated MS (Table 1).

**Table 1:** mRNA expression of genes associated with T-helper (T_H_) cell immune activation and regulatory T-cell activity in CD4+ and CD8+ T-cells from untreated and natalizumab treated MS-patients.

		CD4^+^ T-cells					CD8^+^ T-cells				
		Untreated MS		Natalizumab-treated MS			Untreated MS		Natalizumab-treated MS		
	Targets	n	NR	n	NR	p-value	n	NR	n	NR	p-value
**T_H_1**	***TBX21***	11	0.3 (0.2–0.4)	14	0.2 (0.1–0.4)	NS	10	0.9 (0.4–1.5)	13	1.2 (0.7–2.0)	NS
	***HLX1***	11	0.3 (0.1–0.9)	14	0.3 (0.2–0.5)	NS	8	0.03 (0.0–0.1)	13	0.1 (0.0–0.1)	NS
	***IFNG***	11	0.2 (0.1–0.4)	13	0.2 (0.1–0.3)	NS	10	0.3 (0.3–1.1)	13	0.8 (0.3–1.2)	NS
**T_H_17**	***RORC***	11	2.1 (1.3–2.6)	14	3.0 (1.4–4.8)	NS	10	0.8 (0.5–1.8)	13	1.1 (0.7–3.0)	NS
	***IL17A***	2	0.8 (0.2–1.2)	4	1.8 (1.0–2.3)	ND	0	NA	0	NA	NS
	***IL17F***	1	NA	0	NA	ND	7	2.5 (1.2–3.5)	0	NA	ND
	***IL22***	2	0.4 (0.2–0.8)	0	NA	ND	0	NA	0	NA	ND
**T_H_2**	***GATA3***	11	4.6 (3.5–5.4)	14	4.3 (3.1–5.5)	NS	10	3.0 (2.1–4–3)	13	3.7 (3.6–6.0)	NS
	***IL4***	5	0.03 (0.0–0.1)	6	0.1 (0.0–0.2)	ND	4	0.04 (0.04–0.1)	0	NA	ND
	***IL5***	2	0.5 (0.3–0.5)	0	NA	ND	0	NA	0	NA	ND
**Others**	***FOXP3***	11	2.9 (2.3–3.7)	14	2.1 (1.5–3.0)	NS	10	0.2 (0.2–0.2)	13	0.2 (0.1–0.3)	NS
	***IL10***	9	0.7 (0.5–0.8)	9	0.1 (0.1–0.2)	p<0.001	10	0.5 (0.4–1.1)	10	0.2 (0.2–0.3)	P = 0.002
	***TGFB1***	11	1.1 (0.9–1.3)	14	1.0 (0.6–1.1)	NS	10	1.0 (0.8–1.5)	13	1.2 (0.8–1.4)	NS
	***TNF***	11	0.5 (0.6–1.0)	14	1.3 (1.0–1.5)	P = 0.009	10	0.2 (0.2–0.5)	13	0.6 (0.4–0.7)	P = 0.004
	***PRF1***	11	0.2 (0.1–0.3)	14	0.2 (0.2–0.4)	NS	10	1.1 (0.5–1.4)	13	1.3 (0.7–1.6)	NS

Data are given as median with interquartile range in the parenthesis; statistical analysis was by Mann-Whitney U test and p<0.01 was used as level of significance. When a target was not measurable in more than one sample from a group, data were not applied to the table (NA); when a target was not measured in at least 6 individuals of each group, statistics were not done (ND); NS = not significant.

### Reactivity of myelin-specific CD4^+^ T-cells and non-specifically stimulated CD4^+^ T-cells from untreated and natalizumab-treated MS patients

**Figure 5:**
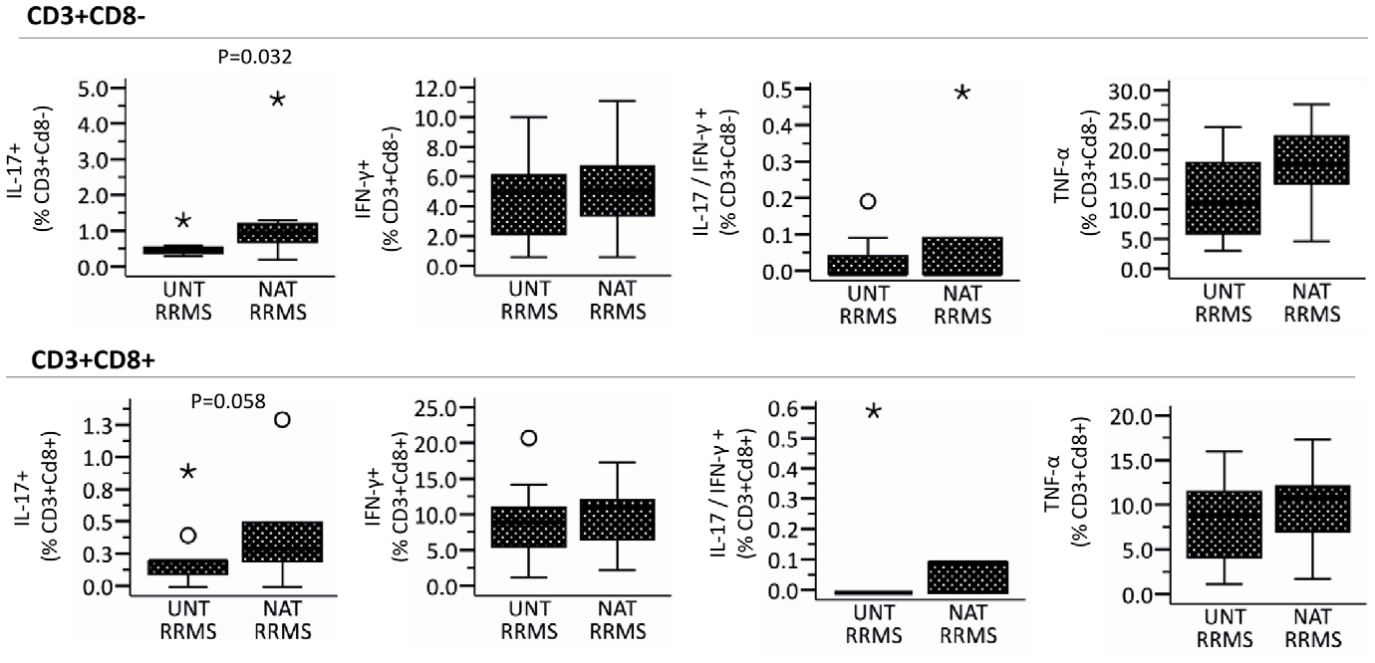
Intracellular cytokine expression in PMA/ionomycin stimulated T-cells from 13 natalizumab (NAT) and 11 untreated (UNT) RRMS patients *ex vivo*. Unstimulated peripheral blood mononuclear cells (PBMCs) were incubated for 7 days as negative control in our CFSE assay and stimulated with PMA/ionomycin for five hours, stained for cell surface markers, permeabilized and stained for intracellular cytokines. The cytokine expression was assessed by flow cytometry as percentage of interleukin (IL) -17^+^, interferon (IFN) -γ^+^, IL-17/IFN-γ or tumor necrosis factor (TNF) -α expressing CD8^−^CD3^+^ and CD8^+^CD3^+^ T-cells. Boxes represent interquartile range, median value indicated as a line, whiskers represent range, ^o^ = outliers, * = extremes. Statistics was by Mann-Whitney U test.

We stimulated PBMCs from untreated and natalizumab-treated MS-patients with MBP and TT. We then assessed the frequency of proliferating CD4^+^ T-cells. We found no differences in the CD4^+^ T-cell proliferation or cytokine expression profile in untreated and natalizumab-treated MS patients *ex vivo* (Table 2). To examine the general potential of CD4^+^ and CD8^+^ T-cells from natalizumab-treated and untreated MS patients to express effector cytokines, we measured the cytokine production in response to PMA/ionomycin in cell cultures that had not been exposed to antigen. Even though our criteria for significance were not met, there was a trend towards a higher frequency of IL17-expressing CD3^+^CD8^−^ T-cells and CD3^+^CD8^+^ T-cells in natalizumab-treated MS patients (Figure 5)

**Table 2:** CD4^+^ T-cell proliferation and cytokine expression in response to myelin basic protein (MBP) and tetanus toxoid (TT) *ex vivo*.

	Myelin Basic Protein				Tetanus Toxoid			
	Untreated MS		Natalizumab-treated MS		Untreated MS		Natalizumab-treated MS	
	n	%	n	%	n	%	n	%
**Fraction of proliferating CD4^+^ T-cells**	13	4.1 (1.4–8)	14	2.75 (2.1–3.6)	12	6.2 (2.7–9.5)	14	9.25 (5.5–16.9)
**IL-17^+^ proliferating CD8^−^ T-cells**	11	10.7 (5.0–21.2)	13	18.5 (7.6–23.8)	11	9.7 (4.8–16.3)	14	6.5 (2.4–14.7)
**IFN-γ^+^ proliferating CD8^−^ T-cells**	11	27.8 (7.7–40.0)	13	29.0 (16.2–41.8)	11	37.2 (32.1–75.5)	14	56.2 (35.4–77.8)
**IL-4^+^ proliferating CD8^−^ T-cells**	11	11.1 (8.7–21.3)	13	11.8 (5.2–20.6)	11	15.3 (11.5–25)	13	18.9 (9.3–26–5)
**TNF-α^+^ proliferating CD8^−^ T-cells**	11	42.0 (27.2–51.1)	13	42.5 (23.6–59.0)	11	46.8 (41.8–64.0)	13	49.3 (38.2–55.4)
**IL-13^+^ proliferating CD8^−^ T-cells**	11	8.0 (5.8–23.2)	11	2 (1.0–13.3)	11	21.7 (13.6–27.8)	11	13.7 (12.6–34.5)
**IL-10^+^ proliferating CD8^−^ T-cells**	11	5.6 (1.8–7.5)	13	4.8 (1.6–10.0)	11	4.1 (1.2–14.4)	13	3.2 (2.0–12.9)

To assess antigen-induced CD4^+^ T-cell proliferation we incubated CFSE labeled peripheral blood mononuclear cells (PBMCs) from untreated and natalizumab treated MS patients with myelin basic protein and tetanus toxoid for 7 days. Using flow-cytometry CD4^+^ T-cell proliferation was assessed as the percentage of CFSE diluted cells within the CD4^+^ T-cell proliferation. After activation with phorbol 12-myristate 13-acetate and staining of intracellular cytokines the percentage of cytokine expressing cells within the proliferating CD8^−^ T-cell population was determined by flow-cytometry. Data are given as median with interquartile range in the parenthesis. There were no significant differences in antigen-specific CD4^+^ T-cell reactivity between untreated and natalizumab treated MS patients using Mann-Whitney U Test with a level of significance p<0.01. IL = interleukin; IFN = interferon; TNF-α = tumor necrosis factor-α.

### Effect of natalizumab on myelin-specific CD4^+^ T-cell responses *ex vivo*

**Figure 6:**
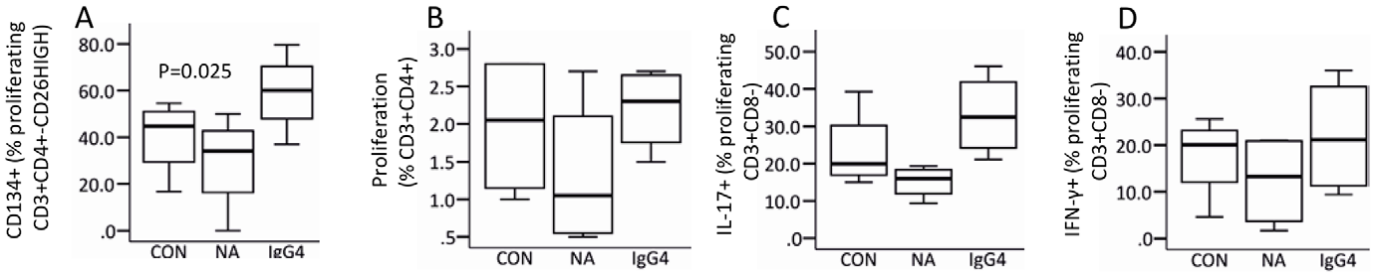
Effect of natalizumab on CD4^+^ T-cell reactivity to myelin basic protein (MBP) *in vitro*. PBMCs were stained with CFSE and cultured for 7 days with MBP (30 µg/ml) and with natalizumab (NA; 25 µg/ml), IgG4 (25 µg/ml) or without further supplement for control (CON). Experiments were done with PBMCs from 4 healthy individuals. After staining for cell surface markers, we assessed (A) CD134 expression on CD26^HIGH^CD3^+^CD4^+^ T-cells, proliferating in response to MBP and (B) proliferation of CD4^+^ T-cells by flow-cytometry. After stimulation with PMA/ionomycin for five hours, staining for surface-markers, permeabilization and staining for intracellular cytokines, the (C) IL-17 and (D) IFN-γ expression of MPB reactive CD4^+^ T-cells was assessed by flow-cytometry. Boxes represent interquartile range, median value indicated as a line, whiskers represent range, ^o^ = outliers, * = extremes. Statistics was by paired sample t-test.

We examined the effect of natalizumab and IgG4-control antibody on the proliferation of CD4^+^ T-cells and their expression profile of CD26, CD134 and intracellular cytokines in response to MBP *ex vivo*. Natalizumab decreased the expression of CD134 on CD26^HIGH^CD4^+^ T-cells which proliferated in response to MBP; the IgG4 control antibody had no significant effect on CD4^+^ T cell reactivity (Figure 6A). Natalizumab had no significant effect on the fraction of proliferating CD4^+^ T-cells (Figure 6B) or on the expression of IL-17A or IFN-γ in MBP-reactive CD3^+^CD8^−^ T-cells (Figure 6C and Figure 6D).

## Discussion

In the present study we show that: 1) decreased VLA-4 surface expression on circulating CD4^+^ and CD8^+^ T-cells in natalizumab treated MS patients is not explained by decreased transcriptional activity of *ITGA4* and *ITGB1*, encoding the α4 and β1 subunits of VLA-4, or associated with decreased expression of LFA-1 integrin. In addition we demonstrate that the observed decrease of VLA-4 surface expression is not caused by competitive binding of natalizumab and the antibody used for detection of VLA-4; 2) numbers of circulating effector memory CD4^+^ and CD8^+^ T-cells are increased in natalizumab treated MS-patients; 3) CD4^+^ and CD8^+^ cells from natalizumab treated patients express increased levels of mRNA encoding TNF-α transcript, however, examination of a broad panel of effector T-cell markers reveals only little evidence for a generally increased activation state of circulating T-cells; 4) reactivity of MBP-specific CD4^+^ T-cells from natalizumab-treated and untreated MS-patients does not differ *ex vivo* but natalizumab-treated patients have decreased expression of CD134 on CD26^HIGH^ CD4^+^ T cells *in vivo* and natalizumab decreases the frequency of MBP-reactive CD4^+^ T-cells that are CD26^HIGH^CD134^+^ *in vitro*.

Explorative studies, as our own, may yield false positive results due to mass significance. We tried to meet this problem by choosing a 1% significance level and by examining groups of functionally associated targets. Furthermore we compared small cohorts which may limit the ability to show significance in some differences we observed. Our T cell reactivity assay is biased towards measuring HLA class II-dependent responses, induced by whole protein preparations, rather than CD8^+^ T-cell responses, as the latter are better studied by analyzing the reactivity to HLA class I-binding peptides. Data interpretation is hampered by differences in disability (EDSS) and disease duration between the group of untreated and natalizumab treated MS patients. Nonetheless, the major positive findings did not correlate with the duration of the MS disease or the disability of the MS patients. There may also be a selection bias regarding the disease course: natalizumab treated patients must have had a history of high disease activity, as this is a requirement to start treatment with natalizumab, whereas the disease course of the mainly newly diagnosed untreated MS patients cannot be predicted. A longitudinal study design had been desirable but was not realizable as most natalizumab treated patients, according to treatment guidelines, receive immunomodulatory treatment with glatiramer acetat or interferon-beta until the day natalizumab-treatment is initiated.

In MS the role of the circulating effector memory T cells, which was the memory T-cell subset that was most profoundly increased in natalizumab treated patients, is uncertain. Treatment with fingolimod, which mainly results in the retention of central memory T cells in lymph nodes, is an efficacious MS therapy [25]–[27]. Indeed, one study has indicated that it is mainly CCR7^+^ T cells that enter the CNS from the blood stream in MS, and that local reactivation within the CNS is needed for the differentiation of these cells to effector memory cells that subsequently invade the CNS parenchyma [16]. Nevertheless, effector memory T-cells have a high capacity to produce pro-inflammatory cytokines [14], [15] and may contribute to an enriched pool of pro-inflammatory cells.

In natalizumab-treated patients, increased frequencies of systemic T-cells and PBMCs that produce pro-inflammatory cytokines such as IL-2, IFN-γ, TNF-α and IL-17 were found, but often only transiently [10], [12]. Even though statistical significance was not reached, our own and another study also showed trends towards increased frequencies of pro-inflammatory, IL-17 secreting, cells in patients treated with natalizumab [5]. In the present study the *ex vivo* response of CD4^+^ T-cells to MBP did not differ between natalizumab-treated and untreated MS-patients. This argues against a selective accumulation of potentially auto-aggressive MBP reactive CD4^+^ T-cells in the circulation in natalizumab-treated MS patients. However, we cannot exclude that other pathogenic auto-reactive T-cells may be increased. Furthermore, it is uncertain to what extent the CFSE assay used in the present study measures antigen-reactivity in central memory and effector memory T cell subsets [14], [15].

Other explorative studies suggested that expression levels of mRNA encoding transcription factors and cytokines associated with T-cells are increased in circulating immune cells in natalizumab-treated patients. One study found transiently increased expression of *IL17* and *CCR6* and persistently increased expression of *IFNG* in circulating CD4^+^ T-cells from natalizumab treated patients; *TNF*, *TGFB*, *TBX21* and *RORC* expression was not affected. Another study found increased expression of *TNF* and *IFNG* but not *IL23*, *IL17A*, *IL17F*, *IL13*, *IL10*, *IL4* and *TGFB* in PBMCs from natalizumab-treated patients [10], [11]. However, as *TNF* and *IFNG* are expressed in many blood cell subsets, conclusions on the activation state of T-cells cannot be drawn from the latter study. We examined transcriptional activity of a broad panel of genes associated with T_H_1/T_H_2/T_H_17 cells and T-cell activation in circulating T-cells and in whole blood samples and found an increased expression of *TNF* (broadly expressed in many cell types including T_H_1 and T_H_17 cells) and decreased expression of *IL10* in CD4^+^ and CD8^+^ T-cells. Taken together, our and other gene-expression studies, measuring cytokines and T-cell-polarizing transcription factors in circulating immune cells from natalizumab treated MS patients, are inconsistent and not obviously suggestive of increased T-cell activation in vivo.

CD161 is a transmembrane cell surface molecule which is expressed on CD4^+^ and CD8^+^ T-cells, and a single nucleotid polymorphism of the CD161 gene (*KLRB1*) has been associated with susceptibility to develop MS [21]. In MS, surface expression of CD161 is increased on circulating CD8^+^ T-cells, and CD161^+^ CD8^+^ T-cells are present in MS lesions. How CD161 potentially contributes to the pathogenesis of MS is uncertain [28]–[29]. All major IL-17 producing T-cell subsets express CD161 [20]. In agreement with this we found, compared to CD161^NEG^ cells, higher expression of the IL-23-receptor (IL-23R) on CD161^+^CD4^+^ T-cells and on CD161^LOW^CD8^+^ T-cells. Still, CD161 is not a T_H_17-marker, as CD161^+^ T-cells' production of IFN-γ by far exceeds that of IL-17, and in particular CD161^HIGH^CD8^+^ T-cell in MS patients are sensitive to IL-12-induced proliferation and IFN-y production [28]. This again is consistent with significantly higher CD212 (IL-12 receptor-beta 2 chain) expression on CD161^HIGH^ CD8^+^ T-cells as compared to CD161^NEG^CD8^+^ T-cells in the present study. We assumed that assessment of IL23R and CD212 on CD161-subsets could reveal differences in the activation state of CD4^+^ and CD8^+^ T-cells. However, expression of both receptors was comparable in untreated and natalizumab-treated patients.

The co-stimulatory molecule CD26 (dipeptidyl peptidase IV) is expressed on memory CD4+ and CD8+ T cells [17], [18]. Myelin basic protein (MBP)-specific T-cell-clones, derived from MS patients express high levels of CD26 [30], and T-cells with high levels of CD26 express IFN-γ and TNF-α [31]. Increased frequencies of circulating CD4^+^ T-cells and CD8^+^ T-cells, expressing CD26 have been associated with clinical and MRI measures of disease activity in MS [31]–[34]. CD134 is expressed on activated CD4+ T cells (including T_H_ 1, T_H_ 2 and T_H_ 17 and T_REG_ cells) and CD8^+^ T-cells [19] and appears to promote activation, clonal expansion and cytokine secretion of these cells, when challenged with antigen [19], [35]. Stronger CD134 expression on CD26^HIGH^ than on CD26^NEG^ and CD26^INT^ CD4^+^ T-cells suggests that the expression of these molecules may be associated. In a study of IFN-β treatment effects in RRMS, we found that treatment with IFN- β reduced the expression of CD134 on CD26^HIGH^ CD4+ T cells [36], in accordance with what we found in natalizumab-treated patients in our study. Thus decreased CD134 expression on CD26^HIGH^ CD4^+^ T-cells may reflect a decrease in T-cell activation. Indeed, we also found that the expression CD134 was reduced on CD26^HIGH^ CD4^+^ T-cells that proliferated in response MBP in the presence of natalizumab *in vitro*. Depending on the epitope specificity, different VLA-4 antibodies are able to block or stimulate VLA-4 mediated co-stimulatory signals in non-specifically or MBP-activated (CD4^+^) T-cells [1], [2], [37]–[41]. Likewise, natalizumab may prevent VLA-4 engagement by endogenous costimulatory molecules in our system. Overall there is little evidence that natalizumab itself has a pro-inflammatory effect on PBMCs and CD4^+^ T-cell responses *in vitro* [6], [12], [13].

In conclusion our study suggests that effector memory CD4^+^ and CD8^+^ T-cells but not MBP-reactive CD4^+^ T-cells are selectively enriched in natalizumab treated patients. Our own data and other previous publications on the activation state of CD4^+^ and CD8^+^ T-cells are ambiguous and indeed we showed that the T-cell activation marker CD134 is expressed significantly lower on CD4^+^CD26^HIGH^ T-cells in natalizumab-treated RRMS. Our finding that natalizumab also decreased the expression of CD134^+^CD26^HIGH^ which proliferated in response to MBP *in vitro* suggests that natalizumab may not only affect the recirculation of CD4^+^ T-cells but may also directly induce phenotypical changes of the CD4^+^ T-cell. The relevance of this finding for the clinical efficacy of natalizumab remains to be established.

## Supporting Information

Table S1 **Targets used in gene expression analysis by PCR.**
(DOCX)

Table S2 **Specifications of the antibodies and other reagents used to stain cells for flow cytometry.**
(DOCX)
