# Supplementary material for: Effect of Natalizumab on Circulating CD4+ T-Cells in Multiple Sclerosis
Source: PLoS One. 2012 Nov 30;7(11):e47578. doi: 10.1371/journal.pone.0047578 (PMC3511477; doi:10.1371/journal.pone.0047578)
Supplement: Table S1 — Targets used in gene expression analysis by PCR. (DOCX) [file pone.0047578.s001.docx]

**Table S1.** Targets used in gene expression analysis by PCR.

| **Symbol** | **Number** |
| --- | --- |
| *FOXP3* | Hs00203958_m1 |
| *GATA3* | Hs00231122_m1 |
| *HLX1* | Hs00172035_m1 |
| *IFNG* | Hs99999041_m1 |
| *IL17A* | Hs00174383_m1 |
| *IL17F* | Hs00369400_m1 |
| *IL22* | Hs01574154_m1 |
| *IL4* | Hs00174122_m1 |
| *IL5* | Hs00174200_m1 |
| *IL10* | Hs00174086_m1 |
| *MLN51(CASC3)* | Hs00904832_m1 |
| *RORC* | Hs01076112_m1 |
| *TBX21* | Hs00203436_m1 |
| *TGFB1* | Hs99999918_m1 |
| *PRF1* | Hs00169473_m1 |
| *TNF* | Hs00174128_m1 |
| *UbcH5B (UBE2D2)* | Hs00366152_m1 |
