# Supplementary material for: Effect of Natalizumab on Circulating CD4+ T-Cells in Multiple Sclerosis
Source: PLoS One. 2012 Nov 30;7(11):e47578. doi: 10.1371/journal.pone.0047578 (PMC3511477; doi:10.1371/journal.pone.0047578)
Supplement: Table S2 — Specifications of the antibodies and other reagents used to stain cells for flow cytometry. (DOCX) [file pone.0047578.s002.docx]

**Table S2.** Specifications of the antibodies and other reagents used to stain cells for flow cytometry.

| Specifty | Isotype | Clone | Host | Flurochrom | Provider |
| --- | --- | --- | --- | --- | --- |
| CD3 | IgG1, κ | UCHT1 | Mouse | Pacific Blue | BD Biosciences, Denmark |
| CD4 | IgG1, κ | RPA-T4 | Mouse | PerCP/Cy5.5 | BioLegend, USA |
| CD8 | IgG1, κ | HIT8a | Mouse | PE-Cy7 | BD Biosciences, Denmark |
| IL-17A | IgG1, κ | BL168 | Mouse | PE | BioLegend, USA |
| IFN-γ | IgG1, κ | B27 | Mouse | APC | BioLegend, USA |
| IL-4 | IgG1, κ | 8D4-8 | Mouse | PE | BD Biosciences, Denmark |
| TNF-α | IgG1, κ | MAb11 | Mouse | APC | BD Biosciences, Denmark |
| IL-13 | IgG1, κ | JES10-5A2 | Rat | PE | BD Biosciences, Denmark |
| IL-10 | IgG2a, κ | JES3-19F1 | Rat | APC | BD Biosciences, Denmark |
| Foxp3 | IgG1, κ | 206D | Mouse | AF647 | BioLegend, USA |
| CD26 | IgG1, κ | M-A261 | Mouse | PE | BD Biosciences, Denmark |
| CD134 | IgG1, κ | ACT35 | Mouse | FITC | BD Biosciences, Denmark |
| CD154 | IgG1, κ | TRAP1 | Mouse | APC | BD Biosciences, Denmark |
| CD161 | IgG1, κ | DX12 | Mouse | APC | BD Biosciences, Denmark |
| CD212 | IgG1, κ | 2.4E6 | Mouse | APC | BD Biosciences, Denmark |
| IL23R | IgG2b, κ | 7H8 | Mouse | PE | Lifespan Biosciences, USA |
| CD49d | IgG1, κ | 9F10 | Mouse | APC-Cy7 | BD Biosciences, Denmark |
| CCR7 | IgG2a, κ | 150503 | Mouse | PE | R&D Systems, Denmark |
| CD45RA | IgG2b, κ | HI100 | Mouse | APC | BD Biosciences, Denmark |
| CD27 | IgG1, κ | M-T271 | Mouse | FITC | BD Biosciences, Denmark |
| CD11a | IgG2a, κ | G43-25B | Mouse | FITC | BD Biosciences, Denmark |
| CD18 | IgG1, κ | 6.7 | Mouse | APC | BD Biosciences, Denmark |
|  | IgG2a, κ | MOPC-173 | Mouse | FITC | BioLegend, USA |
|  | IgG2b, κ | MPC-11 | Mouse | APC | BioLegend, USA |
|  | IgG2a, κ | 20102 | Mouse | PE | R&D Systems, Denmark |
|  | IgG1, κ | X40 | Mouse | APC-Cy7 | BD Biosciences, Denmark |
|  | IgG1, κ | MOPC-21 | Mouse | FITC | BD Biosciences, Denmark |
|  | IgG1, κ | MOPC-21 | Mouse | PE | BioLegend, USA |
|  | IgG2b, κ | MPC-11 | Mouse | PE | BioLegend, USA |
|  | IgG1, κ | RTK2071 | Rat | PE | BioLegend, USA |
|  | IgG2a, κ | R35-95 | Rat | APC | BD Biosciences, Denmark |
|  | IgG1, κ | MOPC-21 | Mouse | APC | BioLegend, USA |
| CellTrace™ CFSE Cell Proliferation Kit | | |  |  | Invitrogen, Denmark |
| Live/Dead® Fixable Aqua Cell Stain Kit for 405 nm excitation | | | | | Invitrogen, Denmark |
